# miRNAs from Zebrafish Embryo Extracts Inhibit Breast Cancer Invasiveness and Migration by Modulating miR-218-5p/PI3K Pathway

**DOI:** 10.3390/ijms26083812

**Published:** 2025-04-17

**Authors:** Noemi Monti, Daniele Antinori, Sara Proietti, Aurora Piombarolo, Alessandro Querqui, Guglielmo Lentini, Domenico Liguoro, Michele Aventaggiato, Marco Lucarelli, Andrea Pensotti, Alessandro Giuliani, Marco Tafani, Andrea Fuso, Mariano Bizzarri

**Affiliations:** 1 Department of Experimental Medicine, Sapienza University of Rome, 00161 Rome, Italy; daniele.antinori@uniroma1.it (D.A.); aurora.piombarolo@uniroma1.it (A.P.); alessandro.querqui@uniroma1.it (A.Q.); guglielmo.lentini@uniroma1.it (G.L.); michele.aventaggiato@uniroma1.it (M.A.); marco.lucarelli@uniroma1.it (M.L.); marco.tafani@uniroma1.it (M.T.); andrea.fuso@uniroma1.it (A.F.); 2 System Biology Group Lab, Sapienza University of Rome, 00161 Rome, Italy; saraproietti80@gmail.com; 3 SAFU Laboratory, Department of Research, Advanced Diagnostics and Technological Innovation, Translational Research Area, IRCCS Regina Elena National Cancer Institute, 00144 Rome, Italy; domenico.liguoro@ifo.it; 4 Pasteur Institute, Cenci Bolognetti Foundation, Sapienza University of Rome, 00161 Rome, Italy; 5 Research Unit of Philosophy of Science and Human Development, University Campus Bio-Medico of Rome, 00128 Rome, Italy; andreapensotti@gmail.com; 6 Environment and Health Department, Istituto Superiore di Sanità, 00161 Rome, Italy; alessandro.giuliani@iss.it; 7 CRiN, Center for Research in Neurobiology D. Bovet, Sapienza University of Rome, 00161 Rome, Italy

**Keywords:** embryo morphogenetic factors, microRNAs, tumor reversion, epithelial–mesenchymal transition, miR-218-5p, PI3K, E-cadherin

## Abstract

Herein, we demonstrate that soluble factors extracted from the distinct phases of the development of zebrafish embryos (ZFEs) exhibit a specific miRNA profile. We removed proteins and concentrated miRNAs in different phase-related samples, which we investigated further. We observed that ZFEs modulate miRNA expression in both normal and cancerous breast cells, significantly inhibiting the invasiveness and motility of triple-negative breast cancer cells. Namely, ZFEs reactivate the synthesis of miR-218-5p in cancerous cells, leading to the downregulation of PI3K, which consequently alters the distribution of phosphoinositides (such as PIP2/PIP3). Moreover, the silencing of miR-218-5p abolished the ZFE effects. Restoring a proper PIP2/PIP3 ratio is crucial for promoting the regression of the malignant phenotype. Phenotypic reversion follows the extensive cytoskeleton rearrangement and the re-emergence of E-cadherin/β-catenin complexes. In addition, ZFEs antagonize the Epithelial Mesenchymal Transition (EMT) by modulating several pathways, including the TCTP-p53 axis. Overall, these results show that embryo extracts enriched with fish miRNAs reactivate endogenous miR-218-5p in cancerous cells, which in turn downregulates critical pathways involved in tumor progression and metastasis.

## 1. Introduction

Cancer is usually considered a disease caused by genetic alterations (involving mutations), which confer cells malignant properties. Carcinogenesis is an irreversible process since there is no way to “fix” a mutated gene. However, an increasing body of evidence suggests that tumors can also develop in the absence of mutations [1]. Furthermore, the presence of oncogenic mutations does not automatically cause cancer. Tumor development should be considered as an “adaptive” phenotype resulting from the interplay between intra- and extra-cellular factors [2,3]. Speaking of which, several studies showed that the reprogramming of somatic [4] and cancerous cells is possible [5], even in the case of oncogene presence [6].

Indeed, cancer cells that were reseeded into normal microenvironments or treated with different molecular factors showed the remarkable property of reversion into nonmalignant cells [7]. Cancer cells might be brought under control if their microenvironment is exposed to the influence of embryo/oocyte extracts [8,9,10].

It is well recognized that key embryonic developmental pathways are often reactivated or dysregulated during tumorigenesis and cancer metastasis development (i.e., epithelial-to-mesenchymal transition (EMT)) [11]. During their natural evolution, cancer cells undergo extensive phenotypic reprogramming. This involves the selection of small clusters of cells that recapitulate several embryonic-like features, helping to identify many overlapping characteristics of the molecular signatures of developing embryos and cancer [12,13].

These correspondences become useful for identifying new treatment strategies. Under the influence of embryonic extracts, tumor cells can be driven toward senescence, dormancy or differentiation, with the nullification of malignant traits, namely by abrogating the metastatic process [14,15]. Moreover, some unidentified embryonic factors can induce differentiation and phenotypic reframing [16,17,18].

Remarkably, embryo factors (ZFEs) extracted from zebrafish allow cancer patients to recover sensitivity to chemotherapy [19], while improving the quality of life and offering extended survival [20]. In addition, some randomized clinical trials, carried out on metastatic liver cancer patients unresponsive to conventional treatments, demonstrated a remarkable clinical effect, with a life expectancy exceeding 60% after 40 months and a 2.4% rate of complete regression [21]. Moreover, we show that low-molecular-weight factors obtained from zebrafish embryos can significantly promote apoptosis in colon cancer, inducing tumor reversion by antagonizing EMT [17].

Previous standard analyses of embryo fish extracts do not demonstrate any specific proteins known for anticancer properties [19]. Instead, we hypothesized that the biological effects shared by ZFEs could likely be ascribed to their miRNA composition. Recently, the results of studies in animal models and preclinical studies of solid cancers have confirmed the effectiveness of treatments employing microRNAs, which are able to target specific oncogenic pathways [22]. Given that microRNAs (miRNAs) are known to play a critical role in cell reprogramming and differentiation, namely by inhibiting EMT [23], we wondered if ZFEs could include miRNAs and therefore decided to test this hypothesis.

However, it is still necessary to recognize the mechanism(s) behind the phenotypic reprogramming fostered by embryonic factors. Particularly, considering the reversion as a specific case of “critical transition” between different attractor states (i.e., differentiated phenotypes), we should identify those critical bifurcation points where the system manifests strong responsiveness, even to weak internal/external “control factors” [24].

## 2. Results

*The preparation of zebrafish embryos*. Zebrafish has 70% gene homology with respect to human cells, which increases to 82% when considering genes related to human disease [25]. To investigate miRNA release, collected embryos were distributed into six phases according to their maturation stages, also identified as *hpf* (i.e., hours post-fertilization). In detail, for each stage, 516 zebrafish embryos were kept and distributed as follows—during the blastula period, 30% epiboly (F1, 5 hpf); during the Gastrula period, 80% epiboly (F2, 8 hpf), tailbud (F3, 10 hpf), 10 somite stage (F4, 14 hpf), 18 somite stage (F5, 16–17 hpf), and 20 somite-stage (F6, 19 hpf)—corresponding to the segmentation period, according to the developmental phases of the zebrafish embryos when kept at the standard temperature (10 °C) [26]. Samples for each phase were diluted to obtain final solutions with different protein concentrations (0.1, 0.3, 3, 10, and 30 µg/mL).

**Figure 1:**
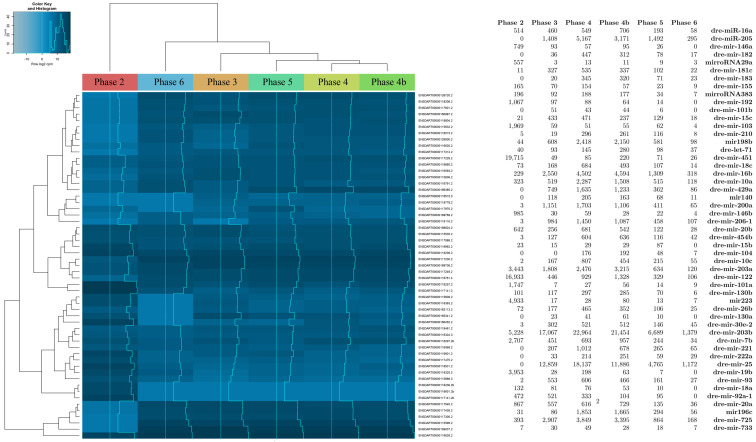
miRNA sequence analysis performed on zebrafish embryos. Data are expressed as raw counts above noise floor.

Embryo samples were then analyzed to determine their miRNAs profile along the developmental process during the first 24 h of development, according to the phase distribution (from F1 to F6). The identified sequences were partitioned into miRNA, RNA, and unknown (i.e., small RNA clones could not be functionally annotated but were mapped to the currently available zebrafish genomic sequences). miRNA sequences were aligned to create the count matrix: both a restricted analysis and a broader analysis were carried out. The restricted analysis allowed us to highlight the sequences that had the best alignment with the zebrafish genome. The identification allowed us to recognize 51 sequences corresponding to 51 microRNAs, as shown in Figure 1.

The statistical distribution of the expression levels of miRNAs in the consecutive phases is summarized in Table 1. We noticed a one-order-of-magnitude temporal variation range between mean miRNAs expressions and a huge variability of expression across miRNAs species, despite an invariant coefficient of variation, with the exception being represented by phase 1. This behavior suggests that the miRNAs dynamics in time are very far from pure stochasticity, reflecting an intrinsic order.

The huge variability across miRNAs reflects the contemporary presence of very low (or absent) and extremely high expression levels, while the constancy of CV suggests a strong correlation among different phase profiles (Figure S1, Supplementary Materials).

This hypothesis is indeed confirmed via the principal component analysis (PCA) applied to the matrix, which has the six different phases as columns (variables) and the 51 miRNA species as rows (statistical units). Table 2 reports the component loading (Pearson correlation coefficients of original variables with extracted components) matrix together with the amount of variance explained by each component [27]. The strong correlation among expression profiles is mirrored by the presence of a first principal component (PC1), explaining 83% of total variance, which suggests that the relative proportions in expression levels remain substantially invariant across time.

The presence of a dominant first component explains that the major part of variance allows the system to acquire a high degree of coherence, while minor components “sustain” the relentless adaptation to endless changes in the microenvironment. The component loading distribution (Table 2) suggests the existence of strictly controlled system dynamics, with Pearson correlations near unity for all the different profiles with PC1. The only remarkable exception is phase 1, corresponding to a different ‘attractor’ dependent on maternally stored mRNAs [28]. miRNAs release from embryo’s cells does not start until mid-blastula in zebrafish [29]. Once the blastula phase has passed, the embryo acquires its own miRNAs competence and “exhausts” the maternally stored miRNAs.

**Figure 2:**
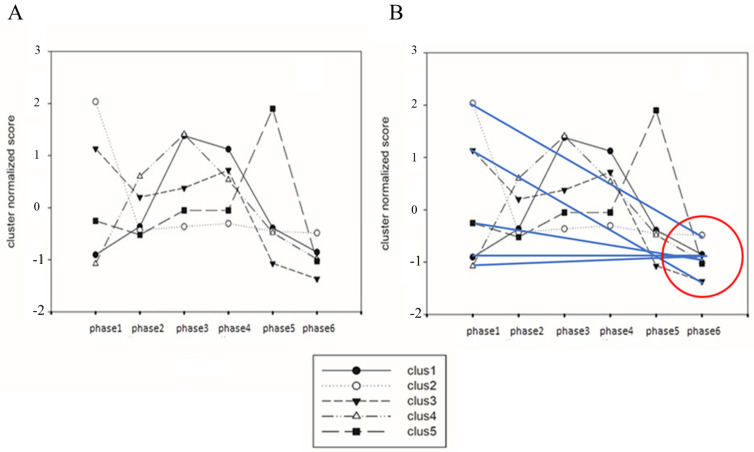
Dynamics of miRNAs are reported (**A**) highlighting how the main clusters (representing 94% of variance as assessed by PCA) converge into a stable attractor (**B**) located at phases 5–6, in which the difference in miRNAs expression is reduced to a minimum value.

We performed a detailed analysis of single-miRNA species over time through a divisive clustering procedure (VARCLUS). These single species are grouped based on the correlation of their temporal trajectories. This procedure creates clusters of miRNAs that move together along phases (maximal within-cluster temporal correlation) and are as de-correlated as possible across clusters (minimal between-cluster correlation) [30]. The data are reported in Figure S1, while the overall dynamics of the miRNAs clusters are shown in Figure 2A. Along the developmental path—from phase 2 to phase 6—we may observe the decay of the initial (phase 1) ‘singular’ profile, which undergoes a progressive expression decrease. The overall expression of all clusters converges towards a “stable state” in phase 5 and 6 (Figure 2B), characterized by minimal differences among the phases in their relative miRNAs expression (with a unique exception represented by a cluster constituted by the single-miRNA 15b species—augmented during phase 5). This behavior is remarkable, as the convergence of several miRNAs clusters indicates the attainment of a stable attractor. Indeed, the developmental stages corresponding to terminal phases (phase 5 and 6) acquire a full differentiating competency [31,32] and this precious property could be conveniently exploited since the organized activity of this network contributes to shaping a “morphogenetic attractor”.

**Figure 3:**
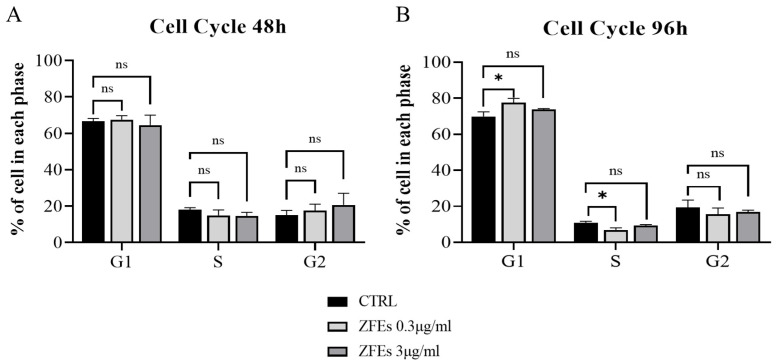
*Effects of 0.3 µg/mL and 3 µg/mL on the cell cycle of MDA-MB-231.* Histograms illustrate the percentage of cells in each phase of the cell cycle. Treatment with ZFEs for 48 h does not affect the cell cycle (**A**). However, after 96 h of treatment, there is a significant decrease in the percentage of MDA-MB-231 cells in the S-phase and a corresponding increase in the G1-phase (**B**). Data are presented as mean ± SD. Statistical significance was determined using one-way ANOVA followed by Turkey’s multiple comparison test (* *p* < 0.05); ns: no significance.

*Zebrafish extracts (ZFEs) modify the cell cycle in MDA-MB-231 breast cancer cells.* Our previous studies demonstrated that ZFEs significantly reduce proliferation and enhance the apoptosis rate across various cancer cell types [33]. In this study, we assessed the dynamics of the cell cycle using flow cytometry. After 96 h of treatment, ZFEs notably decreased the number of cancer cells in the proliferative phase (S) while increasing the population in the resting phase (G1) (Figure 3).

**Figure 4:**
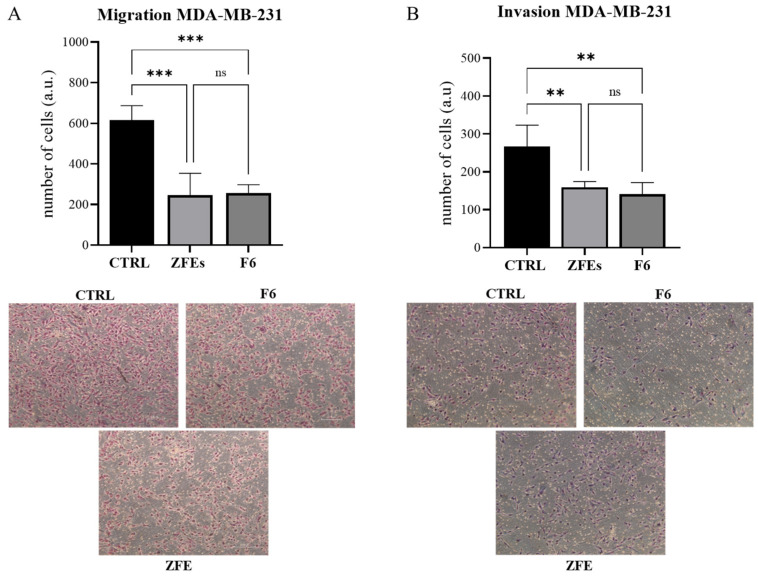
*Effects of 0.3 μg/mL of ZFEs and F6 on migration (**A**) and invasion (**B**) in MDA-MB-231 cells were assessed using Transwell assays*. MDA-MB-231 cells were either untreated (ctrl) or treated with F6 or ZFEs for 24 h. Values represent the number of migrating and invading MDA-MB-231 cells. Histograms show the mean ± SD from three independent experiments performed in duplicate. Statistical significance was determined by one-way ANOVA followed by Tukey’s multiple comparison test (** *p* < 0.01; *** *p* < 0.001). Images were captured using optical microscopy. Scale bars represent 250 μm; ns: no significance.

*Zebrafish extracts (ZFEs) inhibit invasiveness and migrating capability in breast cancer cells.* Both ZFEs and F6 are able to inhibit the migration and invasive capabilities of MDA-MB-231 cells (Figure 4A,B). Specifically, migration decreases (−78% and −61% in ZFEs and F6-treated cells) in MDA-MB-231 cells treated with ZFEs, while invasiveness shows a remarkable reduction, ranging from −45 (ZFEs) to −55% (F6) of values recorded in control samples.

*ZFEs induce significant changes in the miRNA expression pattern in cancerous and normal breast cells*. To assess if treatment with ZFEs could modulate miRNAs release in breast cells, we investigated the overall miRNAs expression in MDA-MB-231 and MCF10A cells, 24 h after the addition of either ZFEs or F6 at a concentration of 0.3 μg/mL.

**Figure 5:**
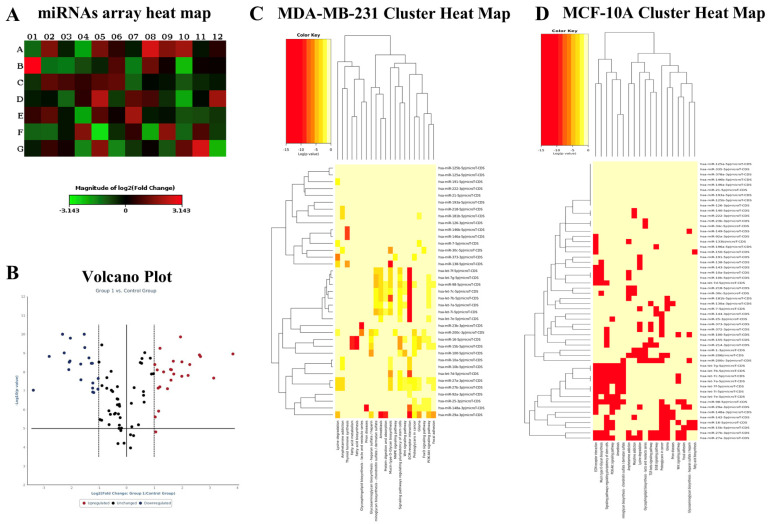
Array analysis of miRNAs. Heat map (**A**) and volcano plot (**B**) show that ZFEs treatment modulates miRNAs expression. Cluster analysis (**C**,**D**) evidenced the differences between the two cell lines.

Array analysis of miRNAs shows that ZFEs modulate a selected number of miRNAs in both MDA-MB-231 and MCF10A cells. However, relevant differences emerge between the two cell lines (Figure 5). Namely, the array shows a trend of increasing for the miRNAs *let-7a*, *218-5p*, and *373* in MDA-MB-231 cells, while in MCF10A only miR-*218-5p* and *373* are upregulated (Figure S2). To confirm such preliminary results, we performed an RT-PCR assay. We confirmed that only miR-*218-5p* was upregulated by both ZFEs and F6 in cancerous and normal breast cells. Similarly, the increase in miR-*373* was only present in MDA-MB-231 cells. No significant differences were recorded between ZFEs and F6 treated cells. Therefore, in the subsequent investigation, we only utilized the mixture (ZFEs) comprising all the phases.

**Figure 6:**
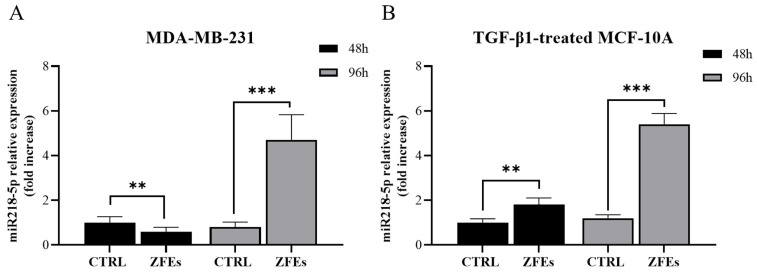
*miR-218-5p expression*. The transcription of miR-218-5p was analyzed in MDA-MB-231 (**A**) and MCF-10A (**B**) cells following treatment with ZFEs. Real-time PCR analysis was performed on miR-218-5p in both cell lines, with MDA-MB-231 (**A**) and MCF-10A (**B**) pre-treated with TGF-β1, at 48 (black) and 96 (light gray) hours of culture. A significant increase in miR-218-5p expression was observed after 96 h of ZFEs treatment in both cell lines. Data are presented as mean ± SD. ** *p* < 0.01; *** *p* < 0.001.

*The focus on miR-218-5p*. Among the miRNAs that showed significant modulation, miR-218-5p was found to be overexpressed (~5-fold) in both cell lines treated with ZFEs after 96 h (Figure 6A,B). Notably, miR-218-5p exhibited a transient upregulation in both cell lines at the 24 h mark.

**Figure 7:**
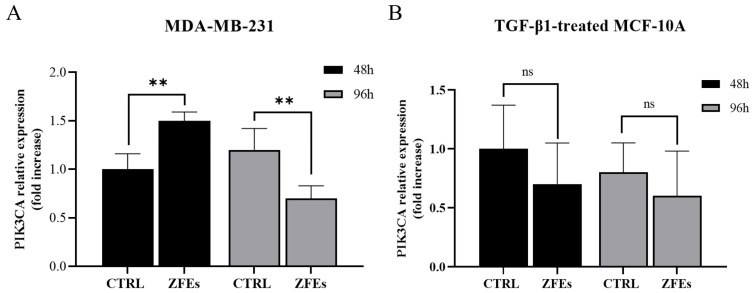
*PIK3C2A expression*. The transcription of PIK3C2A was analyzed in MDA-MB-231 (**A**) and in TGF-β1-pre-treated MCF-10A cells (**B**) at 48 (black) and 96 (gray) hours of treatment with ZFE 0.3 μg/mL. A significant decreased expression of PIK3C2A in MDA-MB-231 was observed after 96 h of ZFEs treatment compared to 48 h. A lower expression of PIK3C2A in MCF-10A cells was observed upon ZFEs treatment, although this was without statistical significance. Data are represented as the mean value ± SD. ** *p* < 0.01; ns: no significance.

*miR-218-5p significantly inhibits PIK3C2A*. PIK3C2A is a specific target of miR-*218-5p*, and miR-218-5p significantly inhibits PI3K expression [34,35]. PIK3C2A mRNA levels are unaffected in MDA-MB-231 cells at 48 h when miRNA-218-5p is still downregulated, while at 96 h, when miR-*218-5p* steadily increases upon ZFEs treatment, mRNA levels of PIK3C2A decrease significantly (Figure 7A). In TGF-β1-pre-treated MCF10A cells, we observed a trend towards a PIK3C2A downregulation in association with miR-*218-5p* overexpression, but the difference fails to reach the level of clear statistical significance (Figure 7B).

**Figure 8:**
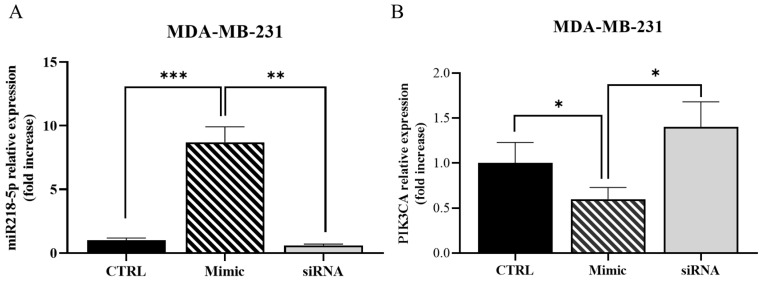
*miR-218-5p transfection*. Real-time PCR analysis of miR-218-5p (**A**) and its target PIK3C2A (**B**) in MDA-MB-231 transfected with a Mimic or an antisense (siRNA) miR-218-5p oligos. A direct correlation was demonstrated between the miR218-5p levels and the expression of PIK3C2A mRNA. Data are represented as mean value ± SD. * *p* < 0.05, ** *p* < 0.01, *** *p* < 0.001.

To validate the direct correlation between miR-218-5p expression and effects on its main target PIK3C2A, MDA-MB-231 cells were transfected with miR-*218-5p* mimic and silencing constructs. Figure 8 shows that the transfection with the mimic miR-*218-5p* sequence results in the robust overexpression of miR-*218-5p* (Figure 8A) and in a significant inhibition of PIK3C2A expression (Figure 8B). Conversely, the transfection assays with the miR-*218-5p* siRNA sequence resulted in significant miR-*218-5p* downregulation (Figure 8A), with the consequent upregulation of its specific target PIK3C2A (Figure 8B).

*ZFEs significantly modulate the PIP2/PIP3 distribution pattern*. The loss of migrating capabilities suggests that ZFEs must significantly modify the distribution and balance of phosphoinositides in the cell membrane. Phosphoinositide 3-kinase plays a key role in chemotaxis, regulating cell motility and invasiveness, mostly through changing the balance between Phosphatidylinositol 4,5-bisphosphate (PtdIns(4,5)P2 or PIP2) and Phosphatidylinositol (3,4,5)-trisphosphate (PtdIns(3,4,5)P3 or PIP3), and, in sequence, by interfering with different downstream signaling components [36,37].

**Figure 9:**
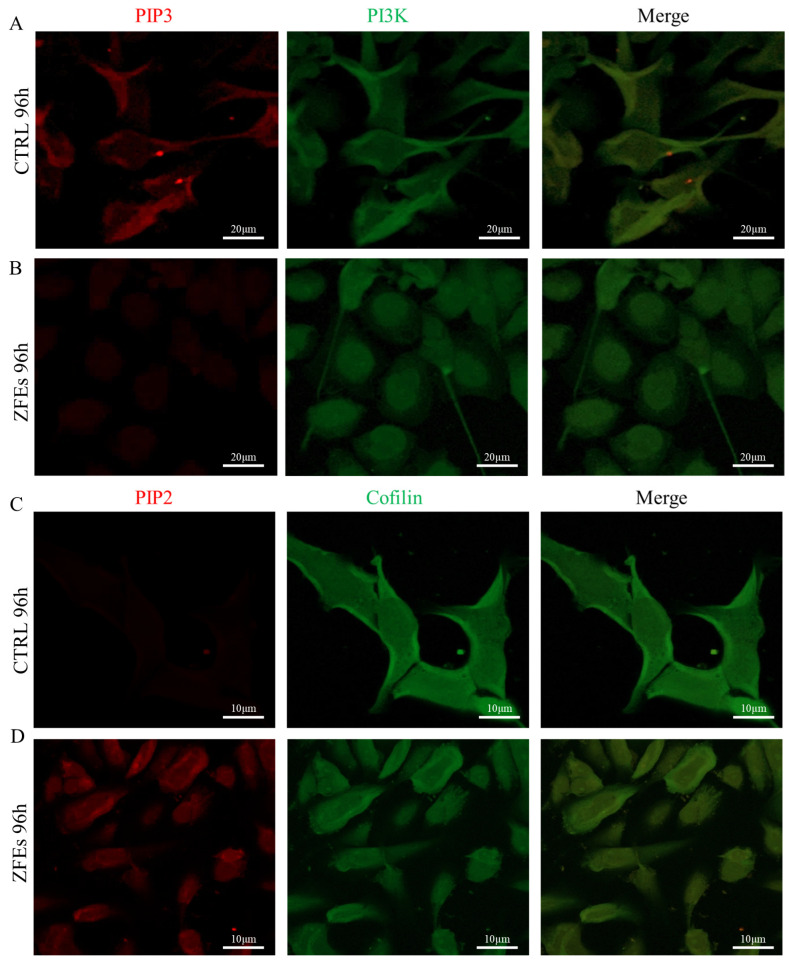

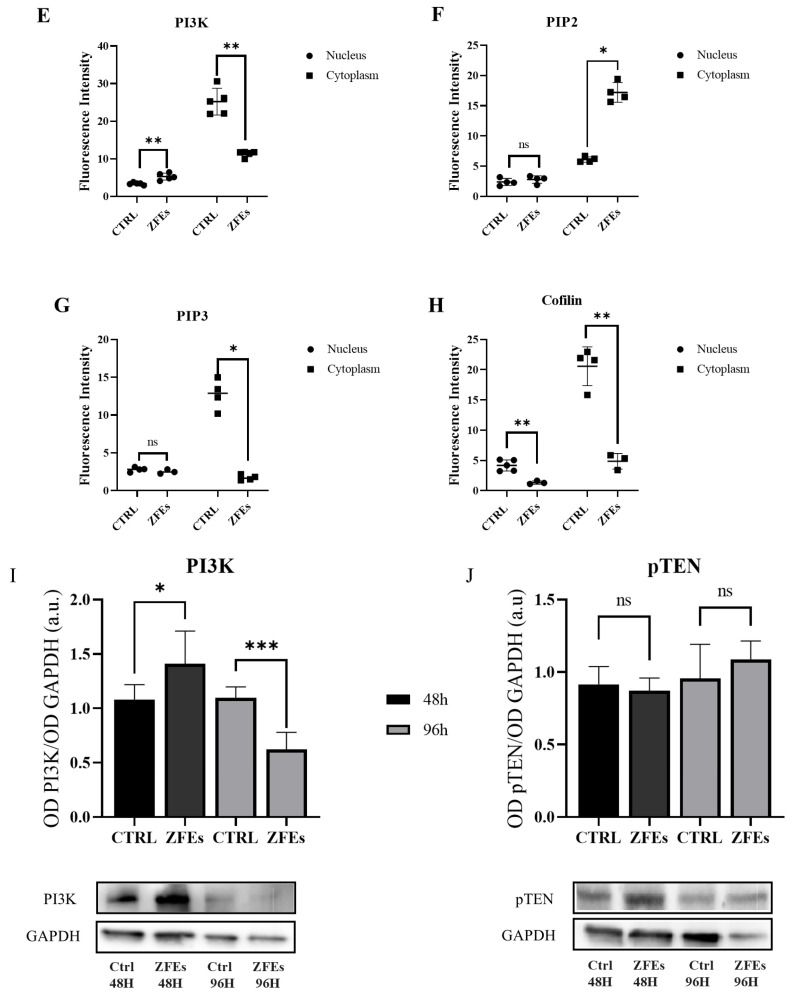
(**A**,**B**) Representative confocal images depicting PIP3 (red) and PI3K (green) in MDA-MB-231 cells cultured under control conditions and after 96 h of ZFEs treatment. In control cells, PI3K was localized in the cytosol, positioned just behind the cell membrane, and exhibited co-localization with PIP3, suggesting the strong enzymatic activity of PI3K (**A**). In contrast, ZFEs treatment resulted in the perinuclear localization of PI3K, with the PIP3 signal nearly disappearing (**B**). Scale bars: 20 μm (**C**,**D**) Representative confocal images of PIP2 (red) and Cofilin (green) in MDA-MB-231 cells under control conditions and following 96 h of ZFEs treatment. Control cells displayed a dense distribution of Cofilin beneath the cortical ring, aligning with the F-actin pattern (**C**). After ZFEs treatment, Cofilin became dispersed throughout the cytosol of MDA-MB-231 cells. Additionally, an increased accumulation of PIP2 at the cell membrane, co-localizing with Cofilin, occurred (**D**). Scale bars: 10 μm. (**E**–**H**) *Fluorescence intensity analysis from nucleus and cytoplasm of PI3K, PIP2, PIP3, and Cofilin in MDA-MB-231 cells*. Fluorescence intensity was evaluated with ImageJ Software. A significant reduction in PI3K cytoplasm signal intensity was observed following treatment with ZFEs. In treated cells, PIP2 cytoplasm signal intensity increased, while the availability of Cofilin markedly decreased, both in the cytoplasm and in the nucleus. Moreover, PIP3 signal markedly decreased in the cytoplasm of treated cells. Data are presented as mean ± SD. Statistical analysis was performed with an unpaired two-tailed Student’s *t*-test. * *p* < 0.05; ** *p* < 0.01. (**I**,**J**) Western blot assay of PI3K (**I**) and PTEN (**J**) protein expression in MDA-MB-231 cells treated with 0.3 µg/mL ZFEs for 48 and 96 h. The analysis corroborated the data obtained from real-time PCR, revealing the decreased protein expression of PI3K in MDA-MB-231 cells after 96 h of ZFEs treatment. Analysis of PTEN expression showed no statistically significant difference following ZFEs treatment. Each sample was normalized against the relative expression of GAPDH. Data are presented as mean ± SD. * *p* < 0.05; *** *p* < 0.001.

Therefore, we investigated the dynamics of PIP2/PIP3 as well as the redistribution of key factors involved in the migratory process—integrins, Cofilin, F-actin, and E-cadherin/β-catenin complexes—in MDA-MB-231 cells. In cancer cells, the PI3K is significantly overexpressed, especially localized in the cytoplasm on the edge of the migrating front (Figure 9A,B). However, after ZFEs treatment, PI3K is significantly decreased in the cytoplasm and increased in the nucleus (Figure 9E). Hence, PIP2, which is deeply reduced in cancer cells, significantly resurfaces after ZFEs treatment, and this occurs markedly in the cytoplasm (Figure 9F), while PIP3 levels significantly drop, specifically in the cytoplasm (Figure 9G). No significant difference has been evidenced in the nucleus levels of both PIP2 and PIP3 in cancer cells treated with ZFEs.

Moreover, we observed a deep decrease in the level of Cofilin in cancer cells upon ZFEs treatment (Figure 9C,D). Specifically, Cofilin dramatically decreases in the cytoplasm and in nuclei of cells after ZFEs treatment (Figure 9H). Particularly, Cofilin is mostly represented behind the cell membrane in treated cells, being tightly associated with PIP2, while in control cells Cofilin is uniformly localized in the cytosol. Overall, the observed modifications involving the PIP2/PIP3 balance are likely a consequence of the decreased PI3K activity, as witnessed by immunofluorescence investigations and further confirmed by Western blot analysis of the PI3K protein at 96 h, when PI3K levels showed values around 50% of those expressed by untreated cancerous cells (Figure 9I). Given that ZFEs treatment does not involve the modulation of PTEN (Figure 9J), we may argue that the downregulation of PI3K expression would suffice in antagonizing those pathways involved in motility and invasiveness downstream of PI3K activation. A relevant role in supporting these changes is likely afforded by the concomitant increase in PIP2, as the experimentally induced increase in PIP2 has been demonstrated to abrogate the metastatic properties of breast cancer cells [38].

**Figure 10:**
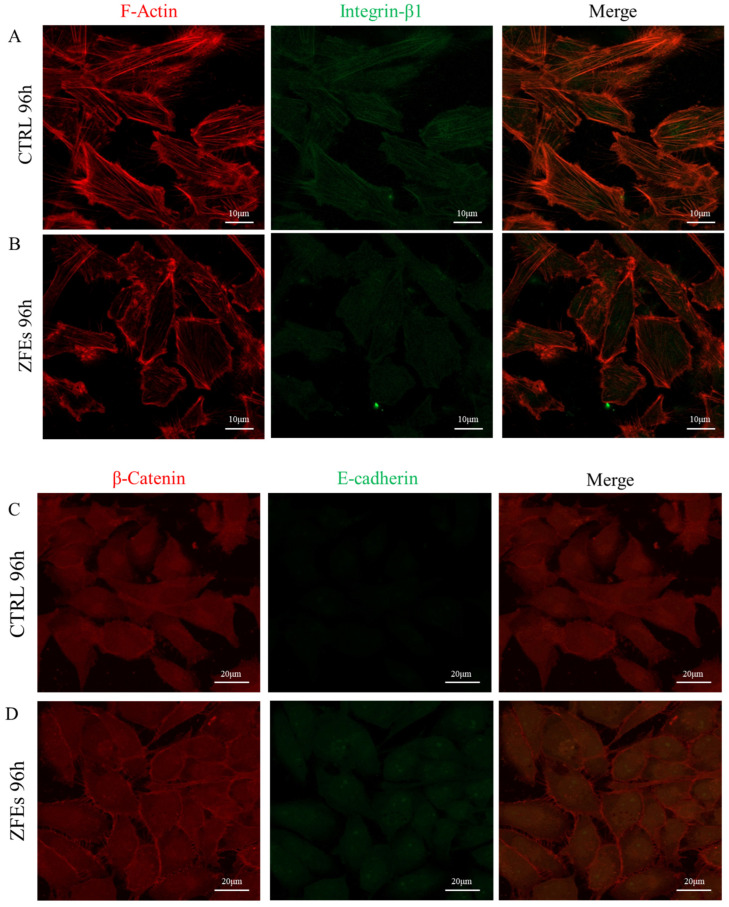
(**A**,**B**) Representative confocal microscopy analysis of F-actin (Red) and integrin-β1 (green) distribution in MDA-MB-231 cells cultured under control conditions or treated with ZFEs for 96 h. Control cells exhibited a dense network of stress fibers (**A**). After ZFEs treatment, F-actin began to reorganize along the cell membrane, with lamellipodia and filopodia being nearly absent (**B**). Scale bars: 10 μm. (**C**,**D**) Representative confocal microscopy analysis of β-catenin (red) and E-cadherin (green) distribution in MDA-MB-231 cells cultured in control conditions or treated with ZFEs for 96 h. In untreated MDA-MB-231 cells, β-catenin was primarily localized in the cytosol and E-cadherin was completely absent (**C**). ZFEs treatment promoted the accumulation of β-catenin/E-cadherin complexes at the cell membrane, facilitating cell-to-cell contact and the reconstitution of an epithelial-like architecture (**D**). Scale bars: 20 μm.

*ZFEs modulate F-Actin and promote the reconstitution of E-cadherin/β-catenin complexes.* In ZFEs-treated cells, *stress fibers* almost completely disappeared, while F-actin was highly polarized and prevalently localized behind cell membranes (Figure 10C,D). Furthermore, treated cells recovered an epithelial morphology, losing the fibroblastic-like phenotype displayed by MDA-MB-231 cells. It is worth noting that the resurfacing of PIP2 plays an instrumental role in promoting the cortical actin polymerization [39].

These changes are associated with the reappearance of integrins (Figure 10A,B). Integrins play a critical role in supporting strong adherence to ECM, limiting cell migratory capabilities. Reduced integrin availability—with consequently weakened integrin–ECM relationships—is followed by the disruption of tissue architecture that precedes the development of invasive breast cancer [40]. Recovery of a “normal” integrin expression pattern upon ZFEs treatment may therefore help in strengthening cell adhesion to the substrate, thus limiting cells’ migratory capabilities.

Yet, the most impressive results were observed when focusing on E-cadherin/β-catenin complexes. E-cadherin—almost undetectable in control cancerous cells—increases significantly and re-establishes close contact with β-catenin, thus reactivating cell-to-cell contact after ZFEs treatment. On the other site, β-catenin, which spreads all along the cytoplasm of untreated cancer cells, shows a typical distribution underneath the cell membrane upon ZFEs addition. Overall, the administration of embryo extracts allows the recovery of an interconnected distribution of cells in culture, mimicking a tissue-like structure (Figure 10C,D).

**Figure 11:**
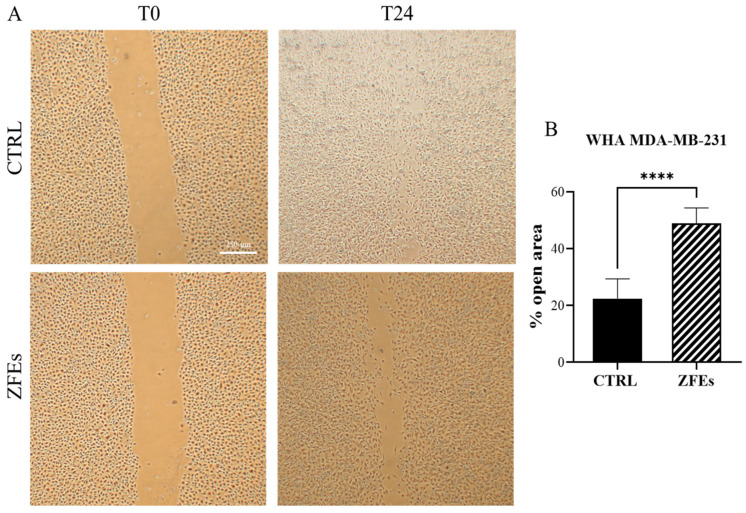
*Wound-healing assay in MDA-MB-231 cells*. (**A**) Representative phase-contrast images of the wound-healing assay taken 24 h after the scratch. The images were captured using an optical microscope. (**B**) Quantitative analysis of wound closure 24 h post-scratch. ZFEs treatment significantly reduced the motility rate of breast cancer cells. Values are expressed as the mean percentage of residual open area compared to the respective cell-free gap at T0. Data are presented as mean ± SD. Statistical analysis was performed using an unpaired two-tailed Student’s *t*-test. **** *p* < 0.0001. Scale bars: 250 μm.

**Figure 12:**
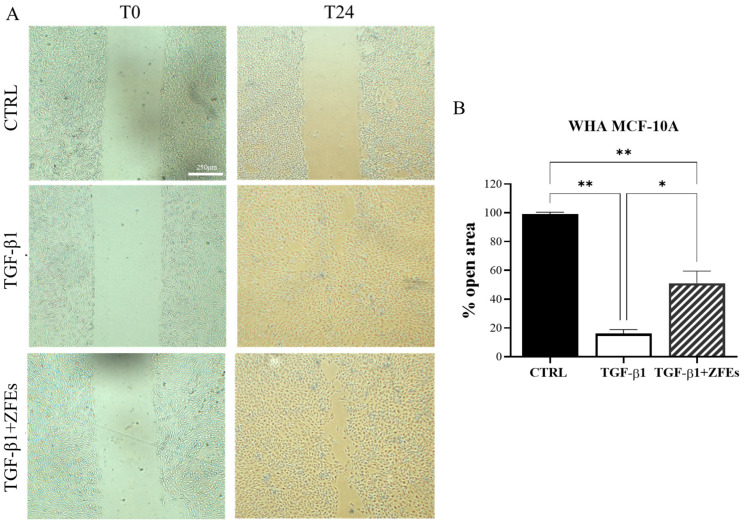
*Wound-healing assay in MCF-10A cells*. (**A**) Representative phase-contrast images of the wound-healing assay taken 24 h post-scratch. Images were obtained via optical microscopy. (**B**) Quantitative analysis of wound closure after scratch. Normal breast cells, upon TGF-β1 treatment, exhibited migratory properties, almost completely filling the scratch area. However, ZFEs treatment reduced the migratory rate, with more than 50% of the area remaining open. Values are expressed as the mean percentage of residual open area compared to the respective cell-free gap at T0. Data are presented as mean ± SD. Statistical analysis was conducted using one-way ANOVA followed by Tukey’s multiple comparison test. * *p* < 0.05; ** *p* < 0.01. Scale bars: 250 μm.

*ZFEs significantly impair the migratory capability of breast cells.* The remodeling of the aforementioned factors is instrumental in modulating the migratory capability of breast cells, as confirmed by the wound healing process investigated in MDA-MB-231. As reported in Figure 11A,B, ZFEs treatment remarkably reduced the migrating abilities of MDA-MB-231. After 24 h, in ZFEs-treated cells, 40% of the scratched area is still open when compared to control cells. We obtained similar results for a wound healing assay performed in MCF-10A cells upon stimulation with TGF-β1. After 5 days of continuous TGF-β1 stimulation, normal breast cells acquire a sustained migratory and invasive phenotype, which constitutes a hallmark of the inflammatory state triggered by TGF-β1. As reported in Figure 12A,B, 50% of the scratched area was still open in ZFEs treated MCF-10A cells, whereas control breast cells almost completely filled the open space.

**Figure 13:**
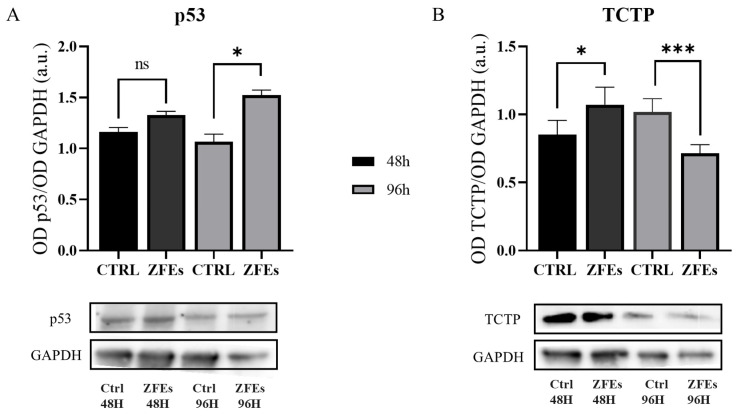
Graphs illustrating the relative expression of p53 (**A**) and TCTP (**B**) in MDA-MB-231 cells treated with 0.3 µg/mL ZFEs for 48 and 96 h. The Western blot analysis highlighted a direct correlation between p53 and TCTP expression. After 96 h of ZFEs treatment, p53 protein levels increased, while TCTP levels significantly decreased. Each sample was normalized against the relative expression of GAPDH. Data are presented as mean ± SD. * *p* < 0.05; *** *p* < 0.001.

*ZFEs induce the relevant modulation of key molecular parameters*. We hypothesized that the abrogation of critical hallmark of malignancy would be associated with modifications in some key molecular parameters, like p53 and TCTP. An increase in p53 expression is a mandatory step in restoring a stable, non-cancerous phenotype, especially when associated with the downregulation of TCTP. Translationally Controlled Tumor Protein (TCTP) is a critical regulator of cell fate commitment and participates in the regulation of pivotal processes, which converge to promote the acquisition of the neoplastic phenotype. TCTP usually increases in cancer, while the downregulation of TCTP is instrumental in promoting cancer reversion [41,42]. In our model, ZFEs significantly upregulate p53 and downregulate TCTP expression at 96 h (Figure 13A,B). Note that these changes appear at a later phase, once cell membrane rewiring and phosphoinositides redistribution are almost complete. Therefore, we can argue that subsequent changes in p53 and TCTP provide support to the transition promoted by ZFEs, rather than being the cause thereof.

## 3. Discussion

Cancerous cells are organized into a hierarchy of genetically and phenotypically heterogeneous subpopulations that can retrieve a “normalized” phenotype under the combined effect of genomic intrinsic dynamics and biophysical pressure exerted by the microenvironment and neighboring cells. Several studies have demonstrated that phenotypic reversion with functional “normalization” can be obtained with pharmacological and biophysical manipulations [43,44]. Particularly, some miRNAs have been demonstrated to play a relevant role in triggering tumor reversion, inhibiting cell growth, promoting apoptosis, and counteracting EMT [45,46,47,48].

It is noteworthy that soluble factors obtained from zebrafish embryos successfully impair cell proliferation, enhance the cancer response to chemotherapy [49], and reverse EMT in neoplastic breast cells [33].

The findings reported herein provide insights into the mechanisms behind these effects. First, we demonstrated that only some ZFE fractions, corresponding to specific developmental stages, exert appreciable biological effects. This result aligns with previous reports [50,51], which evidenced that only embryo extracts obtained from the somite stage—between 14 and 19 *hpf*—can successfully inhibit the cancerous process. Further, we documented that the different phases display a remarkable miRNAs profile, reaching a coherent pattern almost exclusively in fractions #4–6. As anticipated [52], we may confidently suppose that some “reverting morphogens” should be phase-dependent to promote a “phenotypic reconversion” [53]. Such a role can be sustained by miRNAs, given that it has been ascertained that miRNAs establish connections with other miRNAs to achieve regulatory functions, especially during morphogenesis and in mounting adaptive responses to environmental stress [54,55,56]. Indeed, the addition of miRNAs as therapeutic drugs has been already demonstrated to modulate genomic function by modifying the miRNAs of the host. It is well known that miRNAs—from a canonical point of view—control mRNA expression. However, some studies have shown that miRNAs can target non-coding RNAs, including long non-coding RNAs and miRNAs, through a “miRNA–miRNA interaction”, deemed a form of self-regulation.

Therefore, we purified ZFEs by removing almost all of their protein content, leaving only miRNAs specific to each phase in the samples.

ZFEs addition to metastatic breast cancer cells—as well as to breast cells committed to EMT—produced remarkably downregulated invasive capabilities. This effect is associated with a significant reduction in PI3K expression and followed by consequential PIP2 and PIP3 redistribution behind the cell membrane. In turn, the replenishment of PIP2 inhibits Cofilin release into the cytosol, thus preventing the activation of biochemical cascades involved in cell motility. These changes are associated with cytoskeleton remodeling, allowing the cells to regain an epithelial morphology, with the cortical redistribution of F-Actin and the disappearance of *stress fibers*. Finally, upon ZFEs treatment, we observe a significant increase in E-cadherin synthesis, altogether with the re-establishment of E-cadherin/β-catenin complexes. These effects culminate in inhibiting the complex machinery that drives cells toward EMT.

miR-*218-5p* is significantly downregulated in highly invasive breast cancer cell lines [57,58], and the re-expression of miR-*218-5p* inhibits motility and invasiveness in metastatic cells [59]. Moreover, the administration of exosomes enriched in miR-*218-5p* restores miR-*218-5p* levels in breast cancer cells and prevents breast cancer progression, with the simultaneous targeting of angiogenesis and EMT [60]. Therefore, miRNA re-expression therapy could constitute a novel approach to arrest tumor development [61].

We observed that extracts obtained from embryos promoted a significant surge in miR-*218-5p* when added to breast cells. This effect occurred in cancerous and non-cancerous breast cells. In both cases, the increase in miR-*218-5p* was instrumental in fostering a significant decrease in PI3K expression, thus indicating that this mechanism should be included among the physiological roles of miR-*218-5p*.

The activation of PI3K is a pivotal point during EMT in breast cancer [62]. ZFEs reduce PI3K transcription by ~50%, essentially through the increased release of miR-*218-5p*, which specifically targets PI3K. Conversely, silencing miR-*218-5p* abrogates the downregulation of PI3K, thus establishing a mechanistic link between miR-*218-5p* overexpression and PI3K inhibition after ZFEs treatment. Consequently, PI3K modulation results in the modification of several pathways. ZFEs reduce the expression of TCTP and increase p53 expression. This finding is noteworthy, as phenotypic reversion should be properly “constrained” by the contemporary activation of p53 to avoid further unwarranted, pathological issues [63].

Our results highlight the role played by cell membranes in driving the transition process. The plasma membrane plays a pivotal role in orchestrating motility and invasive processes. The cell membrane constitutes a true “dynamic interface”, defining the boundary that mediates the interplay between cells and their microenvironment. Furthermore, the membrane serves for assembling and integrating into countless components of unique platforms, which actively participate in all aspects of the motility process, including force generation, adhesion, and movement regulation [64]. Notably, most of such functions rely on the delicate balance between PIP2 and PIP3, two main phosphoinositides located beneath the cell membrane, where they regulate cytoskeleton organization, motility, ion channels activation, and membrane trafficking [65,66,67].

Remarkably, ZFEs reverse the PIP2-PIP3 pattern behind the cell membrane, as PI3K is the major regulator of the balance between the two. In migrating/invasive MDA-MB-231 and MCF10A cells, both PI3K and PIP3 are overexpressed, while the expression of PIP2—usually the most abundant phosphatidyl-inositol phosphate represented in the plasma membrane [68]—is greatly reduced. On the contrary, after ZFEs addition, the PIP2 distribution beneath the cell membrane steadily increases, while both PIP3 and PI3K are dramatically downregulated. Consequently, Cofilin remains close to PIP2, while in untreated cells, Cofilin spreads all along the cytosol. Cofilin dispersal in cytoplasm is instrumental in activating cytoskeleton remodeling, severing F-actin filaments, and shaping a motile phenotype [69]. The loss of PIP2 leads to an increased release of the membrane-bound pool of Cofilin that can bind to F-actin, favoring its polymerization, as well as lamellipodia emergence, which allows cancer cells to escape tissue constraints, thus becoming metastatic [70]. It is noteworthy that Cofilin is retained at the plasma membrane under resting conditions when PIP2 levels are high, and even a slight reduction in PIP2 density can induce a relevant cytosolic release of Cofilin [71]. Recovering a dense PIP2 distribution is instrumental in inhibiting Cofilin/Actin binding [72] and impairing actin polymerization.

Finally, ZFEs promote a remarkable reconstitution of E-cadherin/β-catenin complexes, which are essential for re-establishing proper cell-to-cell connectivity. The downregulation of E-cadherin and the dissociation of the E-cadherin adhesion complexes are distinctive signatures of increased PI3K activity [73]. Recovering a normal E-cadherin distribution is mandatory for enacting phenotype reversion, as already observed in MCF-10A cells that are committed to acquire an invasive phenotype under the stimulation of TGF-β1 [34]. Conversely, the disruption of E-cadherin-mediated cell–cell adhesion plays an essential role in fostering metastasis [63], while the re-expression of E-cadherin is a signature of the mesenchymal-to-epithelial reversion in MDA-MB-231 breast cancer cells [74]. Moreover, sustained E-cadherin expression is required to drive phenotypic reprogramming in somatic cells [75].

The findings reported herein provide indirect confirmation that cancer cell plasticity can be wisely “manipulated” for therapeutic purposes, enabling cancer cells to “enter” into a less malignant, non-invasive state [76]. This phenomenon is what we refer to as “phenotypic reversion”.

## 4. Material and Methods

### 4.1. miRNA Sequence Analysis on Zebrafish Embryos

The analysis of miRNA content was performed in sequence (Bio-Fab research, Rome, Italy) on six different zebrafish embryo samples (phases 1–6), corresponding to the different stages of the pre-gastrulation times of embryo development. In detail, 516 zebrafish embryo cells were kept for each stage—including the blastula period (F1); the 80% epiboly period (F2); the tailbud period (F3); and the Gastrula period, during which there was a 10 somite-stage (F4), an 18 somite-stage (F5), and a 20 somite-stage (F6)—corresponding to the segmentation period, according to the developmental phases of zebrafish embryo. miRNA analysis was performed as reported in the Supplementary Materials. Zebrafish embryos were kindly provided by Aurora Biosearch Srl, Bollate, Italy.

### 4.2. miRNAs Profiling in Breast Cells

RNA was isolated using the Uneasy Mini Kit (Qiagen, Milano, Italy). Briefly, RNA was isolated using the RNeasy Plus Mini kit (Qiagen, Milano, Italy).

miRNA profiling was performed with Human Cancer Pathway Finder miScript miRNA PCR array (Qiagen, Milano, Italy), containing specific forward primers. The results were elaborated through the “KEGG” and “GO” analyses of the molecular pathways and associated processes using the Diana-miRPath v2.0 software (Qiagen).

Real-time PCR was performed using the CFX Connect Real-Time PCR Detection System (Bio-Rad, Hercules, CA, USA) and SyBr Green (iTaq Universal SyBr Green Supermix, Biorad, Hercules, CA, USA). Overall, 1 μg of total RNA was reverse-transcribed into complementary (cDNA) using the FastGene Scriptase II cDNA kit (Nippon Genetics Europe, Düren, Germany). To obtain miRNA-enriched cDNA, 125 ng of RNA was reverse-transcribed using miScript RTII kit (Qiagen). Then, 1 μg of total cDNA was used for qPCR, utilizing the iTaq Universal SYBR Green Supermix (BioRad, Hercules, CA, USA) for mRNA analysis and the miScript SYBR Green PCR kit (Qiagen, Milano, Italy) for miRNA analysis. mRNA and miRNAs levels were standardized using B2M and RnU6, respectively. The ratio was compared between treated and control groups and the analysis was performed in triplicate for each sample. The results were expressed as the fold change with respect to control values.

U6 forward primer: primers for the miRNAs let7a-5p, 378a-3p, 150-5p, 125b-5p, 373-3p and 218-5p were obtained from the miScript primer assays (Qiagen).

### 4.3. Experimental Cell Model

The breast adenocarcinoma cell line MDA-MB-231 (ECACC Cat# 92020424) was obtained from Sigma-Aldrich (St. Louis, MO, USA), and the non-tumorigenic epithelial cell line MCF-10A (ATCCCRL-10317) was obtained from LGC Standards S.r.l, Milan, Italy. Cells were seeded into 25 cm^2^ flasks (Falcon, Becton Dickinson Labware, Franklin Lakes, NJ, USA). MDA-MB-231 cells were grown in Dulbecco’s modified Eagle’s medium (DMEM) supplemented with 10% fetal bovine serum (FBS) and antibiotics (penicillin 100 IU/mL, streptomycin 100 microg/mL, gentamycin 200 microg/mL—all from Euroclone Ltd., Cramlington, UK). MCF-10A were grown in DMEM:F12 (1:1) medium (Sigma-Aldrich, Merck, Darmstadt, Germany) supplemented with 5% horse serum (Sigma-Aldrich, Merck, Darmstadt, Germany) and 20 ng/mL EGF (Epidermal Growth Factor), 0.5 μg/mL Hydrocortisone, 0.5 mg/mL cholera toxin, and 10 mg/mL insulin (all from Sigma Chemical Co., USA) and antibiotics (penicillin 100 IU/mL, streptomycin 100 μg/mL, gentamycin 200 μg/mL), all of which were from Euroclone Ltd., Cramlington, UK.

Cells were cultured at 37 °C in a 5% CO_2_ atmosphere, and the culture medium was changed every three days. Upon reaching confluence, cells were sub-cultured after removal with 0.05% trypsin—0.01% EDTA. For MDA-MB-231, 0.3 µg/mL ZFEs were added to DMEM, supplemented with 0.1% FBS, for Western blot analysis and RNA extraction. MCF-10A cells were first treated with 10 μM TGF-β1 (PeproTech catalog #100-21) for five days. On the fifth day, 0.3 µg/mL ZFEs or 0.3 µg/mL F6 were added to F12 Ham medium supplemented with 0.5% horse serum.

The ZFEs are composed of one zebrafish embryo for each developmental phase (from phase 2 to phase 6), whereas F6 is composed solely of phase 6 embryos. To extract the content of zebrafish embryos, ZFEs were dissolved in a solving medium (emulsifier in cold phosphate-buffered saline, PBS). Then, we quantified the protein concentration of the solution twice with the Bradford assay. To ensure that the extracts were primarily enriched in terms of smaller molecules—such as miRNAs—the protein content was removed from the ZFEs solution via filtration through a 0.22 µm Millipore filter, effectively removing most of the protein. This was recorded using the Bradford assay. The ZFEs concentration was standardized by considering the protein content before filtration and the number of cells in each developmental phase. The breast cells were then used in an experimental assay to evaluate responsiveness to a concentrated pool of solution composed of fish-derived miRNAs.

### 4.4. Cell Cycle

MDA-MB-231 cells were analyzed under control conditions and after treatment with 0.3 μg/mL and 3 μg/mL of ZFEs for 48 and 96 h. On the day of analysis, the cells were trypsinized and centrifuged, and the pellet was washed twice with PBS. The cells were then fixed with 70% ethanol at 4 °C for 24 h, and stained with PBS supplemented with Propidium Iodide (100 μg/mL, Invitrogen) plus RNaseA (50 μg/mL) for 30 min at 37 °C. Stained cells were measured by flow cytometry. Cell cycle analysis was performed in triplicate.

### 4.5. Cell Migration and Invasion Assay

For the migration assay, 2.5 × 10^4^ cells untreated and treated with 0.3 µg/mL ZFEs, or 0.3 µg/mL F6, were seeded in 500 μL DMEM + 0.1% FBS medium in the upper side of 8 μm filters (Falcon, BD Biosciences, San Jose, CA, USA) (upper chamber) and placed in wells of a 24-well plate (Falcon, BD Biosciences, USA) (lower chamber) containing 800 μL of DMEM 10% FBS medium. After 24 h, the migrating cells on the lower surface of membranes were fixed and stained with Hemacolor^®^ (HX54775574, Merck, Darmstadt, Germany), following the manufacturer’s instructions.

For the invasion assay, 2.5 × 10^4^ untreated cells (CTRL) and cells treated with 0.3 µg/mL ZFEs, or 0.3 µg/mL F6, were seeded in 500 μL of DMEM + 0.1% FBS medium on the upper surface of the transwells containing Matrigel. Transwells were placed in wells of a 24-well plate (lower chamber), containing 800 μL of DMEM 10% FBS medium. After 24 h, Matrigel invasion cells on the lower surface of membranes were stopped, fixed, and stained with Hemacolor^®^ (HX54775574, Merck, Darmstadt, Germany) following the manufacturer’s instructions. To analyze both cell migration and invasion, five images were taken from five different fields for each experimental condition, using the MotiCam S6 camera of the AE 2000 (Motic) optical microscope. We counted the number of cells in each image, using ImageJ software (ImageJ 1.54k Java 13.0.6), under the specified conditions.

For each data point, three independent experiments were performed in duplicate.

### 4.6. RNA Extraction and RT-qPCR

Total RNA isolation from was performed using the RNeasy Plus Mini kit (Qiagen) following the manufacturer’s instructions. The RNA concentration was determined through Nanodrop measurements. An equal amount of total RNA was reverse-transcribed using the iScript cDNA Synthesis Kit (Bio-Rad Laboratories). Quantitative PCR (qPCR) was performed using iTaq Universal SYBR Green (Bio-Rad Laboratories). The PI3KC2A mRNA levels showed in this paper were normalized using GAPDH (Hs_GAPDH_1_SG; Quanti Tech Primer Assay). B2M and 18S were also used as housekeeping genes, giving comparable results with respect to GAPH. The analysis was performed in triplicate for each sample. The PI3KC2A primers used in qPCR are the forward 5′-AGAAGCCATGACGAGACACC-3′ and the reverse 5′-CTGCTCGGTGTTGGTTTTCA-3′. Data are shown as the relative amount versus the control.

### 4.7. miRNA 218-5p Silencing and Overexpression

Transient transfections were performed using Lipofectamine™ RNAiMAX, Invitrogen™ and siRNA/Mimic (MirVana™, Ambion™) in 150 μL Opti-MEM™ (Gibco™) according to the manufacturer’s instructions. We added 250 μL of lipofectamine and siRNA/Mimic at T0 directly in the MDA-MB-231 culture medium and we analyzed the results at 48 h. ON-TARGETplus Non-Targeting siRNA, miRIDIAN microRNA Mimic Negative Control, ON-TARGETplus GAPD Control siRNA (5 nmol, D-001830-01-05), miRIDIAN microRNA Human hsa-miRNA-218-5p (Hairpin Inhibitor 5nmol, IH-300624-06-0005), and miRIDIAN microRNA Human hsa-miR-218-5p (Mimic 5nmol, C-300624-05-0005) were purchased from Dharmacon (Lafayette, CO, USA).

### 4.8. Confocal Microscopy Analysis

Then, 4.5 × 10^3^ MDA-MB-231 cells were cultured into 8-well μ-slides (ibidi GmbH, Am Klopferspitz 19, D-82152 Martinsried, Germany) and treated with 0.3 μg/mL of ZFEs for 96 h. Subsequently, cells were fixed with 4% formaldehyde solution in PBS for 10 min, washed three times with PBS (with Ca^2+^ and Mg^2+^), and permeabilized for 6 min in 0.1 M Tris pH 7.4 and 0.2% Triton^®^ X-100. After three washes in PBS, samples were incubated for 1 h at rt with 0.2 mg/mL BSA, followed by overnight incubation at 4 °C with anti-PIP2 antibody at a 1:100 dilution in BSA 1% (sc-53412 Santa Cruz, Dallas, TX, USA). Cells were then washed three times with PBS and incubated for 1 h with the secondary antibody goat anti-mouse IgG Alexa Fluor^®^ 555 at a 1:200 dilution in BSA 1% (A21429; Invitrogen, Carlsbad, CA, USA). Samples were again washed three times in PBS and the second immunofluorescence assessment was performed with anti-PI3K antibody incubation at a 1:100 dilution in BSA 1% for 3 h at rt (#4249S Cell Signaling C73F8 Danvers, MA, USA), followed by three washes in PBS and incubation using a secondary antibody goat anti-rabbit IgG Alexa Fluor^®^ 488 at a 1:200 dilution in BSA 1% (A11001; Invitrogen, Carlsbad, CA, USA). Finally, after three washes in PBS, samples were mounted using ProLong^®^ Diamond Antifade Mountant (P36961; ThermoFisher Life Technologies, Waltham, MA, USA). The same procedure was performed using the anti-PIP3 antibody (A21328; Invitrogen, Carlsbad, CA, USA) with the secondary antibody goat anti-mouse IgG Alexa Fluor^®^ 555 and using anti-Cofilin antibody (sc-33779 Santa Cruz, Dallas, TX, USA) with the secondary antibody goat anti-rabbit IgG Alexa Fluor^®^ 488. We employed the previously reported dilutions for both primary and secondary antibodies in BSA 1%. Finally, anti-integrin β1 antibody (sc-8978 Santa Cruz, Dallas, TX, USA) was used with the secondary antibody goat anti-rabbit IgG Alexa Fluor^®^ 488 (A11008; Invitrogen, Carlsbad, CA, USA) and Phalloidin TRITC labeled (P1951 Sigma-Aldrich–M. ERCK, St. Louis, MO, USA) was used to bind polymeric and oligomeric forms of actin at a 1:200 dilution in BSA 1% for 30 min at rt. Images were taken using a ZEISS LSM 900 Confocal Microscope (Carl Zeiss Industrielle Messtechnik GmbH, Oberkochen, Germany). The fluorescence intensity was quantified using ImageJ Software. For each experimental condition, ten images were captured and analyzed at 40× magnification. The software enabled the precise quantification of the green and red fluorescent signal by calculating the integrated density based on the corrected total cell fluorescence. We analyzed the signal of fluorescence from the nucleus and cytoplasm of single cells present in each image.

### 4.9. Western Blots

MDA-MB-231 cells were treated with 0.3 μg/mL ZFEs for 48 and 96 h. On the day of experiment, both control and treated cells were washed twice with ice-cold PBS and then scraped into a RIPA lysis buffer (Sigma Aldrich, St. Louis, MI, USA), supplemented with a mixture of protease inhibitors (Complete-Mini Protease Inhibitor Cocktail Tablets, Roche, Mannheim, Germany) and phosphatase inhibitors (PhosStop; Roche, Mannheim, Germany). Cellular extracts were then centrifuged at 8000× *g* for 10 min at +4 °C. Protein content was determined using the Bradford assay. For Western blot analysis, we separated equal amounts of protein for each sample on SDS–polyacrylamide gels and transferred them onto nitrocellulose membranes (BIO-RAD, Bio-Rad Laboratories, Hercules, CA, USA). The following antibodies were analyzed: anti-TCTP (ab37506, Abcam); anti-PI3K (#4249, CST); anti-pTEN (sc-7974, Santa Cruz Technologies, Dallas, TX, USA); and anti-p53 (sc-126, Santa Cruz). Antigens were detected through chemiluminescence with the ECL kit (Western Bright ECL HRP Substrate, Waltham, MA, USA; Immunological Sciences) according to the manufacturer’s instructions.

All Western blot images were acquired and analyzed through the Chemidoc c300 system (Azure Biosystems, Dublin, CA, USA). The optical density (OD) of each condition was normalized vs. the signal of internal control GAPDH (anti-GAPDH #2118 from Cell Signaling Technology, Danvers, MA, USA). Western blot experiments were performed in triplicate.

### 4.10. Wound Healing Assay

To perform the wound-healing assay, MDA-MB-231 cells were seeded in 35 mm petri dishes. Once 100% confluence was reached, a scratch was drawn using a sterile 1000 μL tip. Cells were washed twice with PBS and control cells were fed with DMEM 5% FBS; meanwhile, we treated cells with DMEM and 0.3 μg/mL ZFE for 24 h. The petri dishes were then incubated at 37 °C in an atmosphere of 5% CO_2_. Cells were photographed at 10× magnification with a MotiCam S6 camera of the AE 2000 (Motic) optical microscope(SIAL, Rome, Italy).

A similar wound-healing assay was performed in MCF-10A cells, which were seeded in 35 mm petri dishes. The cells were treated with TGF-β1 for 5 days until full confluence was reached and then scratched with a sterile 1000 μL pipette tip. After washing twice with PBS, control cells were cultured with DMEM:F12 supplemented with 5% horse serum, while treated cells were exposed to either TGF-β1 alone or a combination of TGF-β1 and 0.3 μg/mL of ZFEs. The cells were incubated at 37 °C in a 5% CO_2_ atmosphere. Cells were photographed at 4× magnification using a MotiCam S6 camera of the AE 2000 (Motic) optical microscope. For each experimental condition, ten images were captured at time zero (T0) and then again after 24 h for both MDA-MB-231 and MCF-10A cells. These images were acquired using appropriate imaging techniques, ensuring consistent and reproducible conditions across all samples. Following image acquisition, the images were analyzed using ImageJ software to quantify the area covered by cells at both time points. This analysis allowed for an accurate assessment of the changes in cell coverage over the 24 h period, providing insight into the dynamic behavior of the cells under the experimental conditions given.

### 4.11. Statistical Analysis

The analysis of the fold change in miRNA expression in embryo extracts was performed using the online data analysis tool of Sabosciences (Qiagen). The dynamical changes in miRNA profiles were studied by a multidimensional approach involving both spectral (principal component analysis, PCA) and variable-clustering (VARCLUS procedure) approaches. While PCA gives a global representation of the general invariance of the miRNA profile driven by the existence of stable miRNA profiles (from phase 2), VARCLUS (a clustering procedure creating groups of profiles on the basis of their mutual correlation) [77] allows scholars to obtain a detailed sketch of the (subtle) changes in miRNA profiles across embryo developmental phases.

For Western blot assay, statistical analysis was performed using an unpaired two-tailed Student’s *t*-test. For qPCR, wound healing assay and migration/invasion assay statistical analysis were conducted, using both the unpaired two-tailed Student’s *t*-test and one-way ANOVA, followed by Tukey’s multiple comparison test. All assessments were performed with GraphPad Prism 8 software. Fold-change miRNA expression analysis was carried out using the Sabosciences (Qiagen) data analysis tool. A representation of KEGG pathways was producedwith XLSTAT 2017 (Addinsoft). Data are presented as mean ± standard error of the mean (SEM), with *p*-values of *, *p* < 0.05; **, *p* < 0.01; ***, *p* < 0.001; and ****, *p* < 0.0001 deemed statistically significant.

## 5. Conclusions

Conventional anti-cancer therapies primarily focus on restraining uncontrolled proliferation, overlooking additional oncogenic traits such as phenotypic plasticity or metastatic invasion. The use of PI3K inhibitors as therapy in several kinds of cancer, particularly in breast cancer, faces several challenges due to the frequent mutations in the PI3K signaling pathway. These mutations lead to the sustained activation of the pathway, contributing to tumor progression, cancer cell survival, and the acquisition of drug resistance [78]. The PI3K inhibitors currently in use primarily act as enzymatic inhibitors, targeting the enzyme’s activity to block downstream signaling. However, their effectiveness is often limited by acquired resistance and significant side effects [79]. This highlights the need for alternative therapeutic strategies. Our approach, using zebrafish embryo extracts, differs as it acts as a synthesis inhibitor, potentially targeting the root cause of the dysregulated signaling pathways in cancer cells and offering a novel means of overcoming the limitations of current PI3K-targeted therapies.

Alternative therapeutic strategies—used in association with conventional drugs—can be proficiently envisaged to “re-educate” cancer cells towards a less malignant phenotype. A fraction extracted from embryonic fish and enriched in specific miRNAs could likely belong to this new class of compounds; given that ZFEs upregulate miR-218-5p, it is conceivable that this modification would improve chemotherapy response, as indicated by several studies [80]. Furthermore, the beneficial effects of ZFEs in other cancers—including leukemia, colon, and liver tumors—were observed by several authors, providing evidence that this approach can be effective as a viable therapeutic strategy. The overall evidence suggests that ZFEs improve the quality of life and life survival, while mitigating some deleterious effects of antineoplastic drugs.

## Figures and Tables

**Table 1:** The statistical distribution of the expression levels of microRNAs in the consecutive phases is summarized in Table 1, that reports mean, the standard deviation and coefficient of variation (CV = standard deviation/mean) of the 51 microRNA species during the different phases.

Phase	Mean	Std Dev	CV
1	1341	3700	2.76
2	961	2951	3.07
3	1593	4060	2.55
4	1337	3417	2.56
5	429	1139	2.65
6	93	251	2.70

**Table 2:** The table reported the component loading (Pearson correlation coefficients of original variables with extracted components) matrix together with the amount of variance explained by each component.

Phases/Percent Var, Expl,	PC1	PC2
1	0.103	0.995
2	0.994	−0.02
3	0.997	−0.04
4	0.990	0.004
5	0.998	−0.002
6	0.995	−0.018
Percent Var. Expl.	83	16

## Data Availability

The original contributions presented in this study are included in the article/Supplementary Material. All the data will be available at reasonable request to the corresponding authors.

## Supplementary Materials

The following supporting information can be downloaded at: https://www.mdpi.com/article/10.3390/ijms26083812/s1.
