# Supplementary material for: miRNAs from Zebrafish Embryo Extracts Inhibit Breast Cancer Invasiveness and Migration by Modulating miR-218-5p/PI3K Pathway
Source: Int J Mol Sci. 2025 Apr 17;26(8):3812. doi: 10.3390/ijms26083812 (PMC12027887; doi:10.3390/ijms26083812)
Supplement: Supplementary file 1 [file ijms-26-03812-s001.zip › Supplemental Material.pdf]

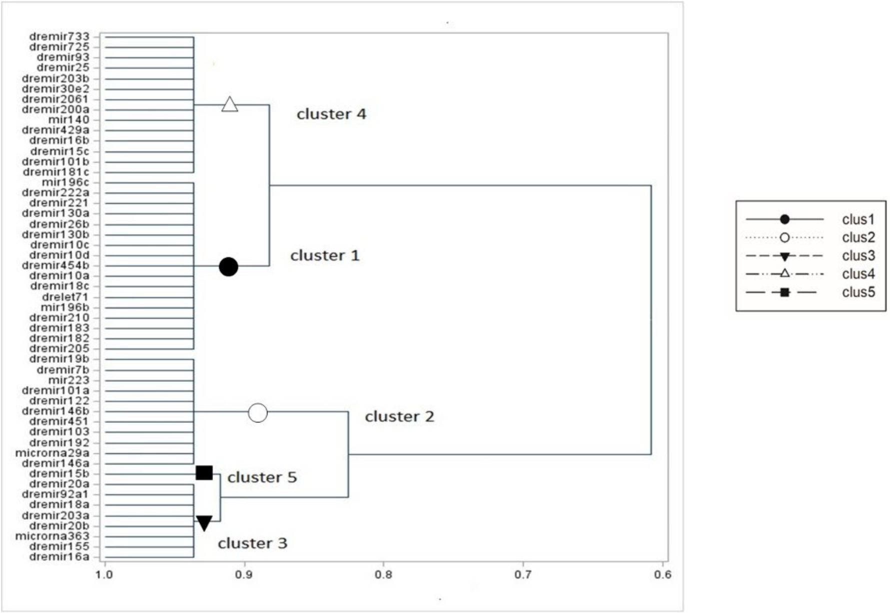

Fig. S1. Abscissa corresponds to the proportion of variance explained by clustering procedure and suggests a five cluster (with one singleton corresponding to dremir15b) solution as the most informative (94% of explained variance) summarizing the whole expression oscillation pattern of the micro-RNA species in time (Fig.2).

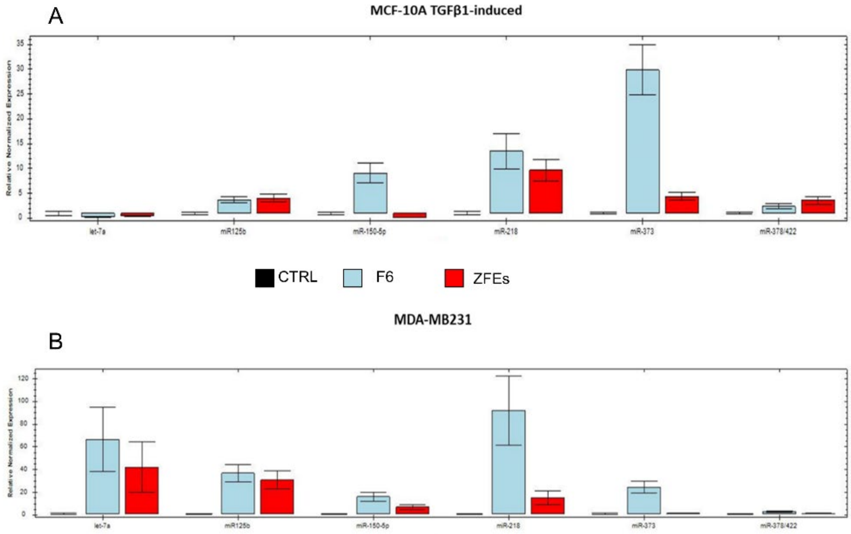

Fig. S2. Expression of miRNAs in MCF10A (A) and MDA-MB-231 (B) cells under treatment with ZFEs or F6 fraction. Histograms indicate the mean value ± SD of three independent experiments performed in duplicate.

Material and Methods

Principle and Procedure

Mature miRNAs are naturally occurring, 22-nucleotide, noncoding RNAs that mediate post-transcriptional gene regulation. Unlike most cellular RNAs, mature miRNAs possess both a 3’ hydroxyl group and a 5’ phosphate group. This allows adapters to be specifically ligated to both the 3’ end and 5’ end of miRNAs enabling universal reverse transcription and library preparation of mature miRNAs, while minimizing the background from other RNA species. In addition, the QIAseq miRNA Library Kit enables library preparation and multiplexing of up to twelve samples using QIAseq miRNA NGS 12 Index IL, up to 48 samples in combination with QIAseq miRNA NGS 48 Index IL or up to 96 samples with QIAseq miRNA NGS 96 Index IL.

Universal cDNA synthesis and library preparation of miRNA

In an unbiased reaction, adapters are ligated sequentially to the 3’ and 5’ ends of miRNAs. Subsequently, universal cDNA synthesis with UMI assignment, cDNA cleanup, library amplification and library cleanup are performed. Proprietary methodology using modified oligonucleotides virtually eliminates the presence of adapter dimers in the sequencing library, effectively removing a major contaminant often observed during sequencing. Additionally, the kit is designed to minimize the presence of hY4 Y RNA, which is often observed in high levels in serum and plasma samples.

Library amplification

Library amplification is accomplished using one of two formats. In format 1, a wet universal forward primer from a tube is paired with 1 of 48 wet reverse primers from tubes (cat. no. 331592 and 331595) to assign each sample a unique index. In format 2, a dried universal forward primer from a plate is paired with 1 of 96 dried reverse primers in the same plate (cat. no. 331565) to assign each sample a unique custom index. In format 2, library amplification reactions occur directly in the index plate, providing a convenient HT indexing solution. The unbiased amplification of all miRNAs in a single reaction ensures that sufficient target is present for next-generation sequencing.

Library cleanup

After library amplification, a cleanup of the miRNA library is performed using a streamlined magnetic bead-based method. Input: 100ng RNA. Sequencing Platform: HiSeq 3000 1x75 (SE) (further detail in Suppl. Mat.)
